# Genetic individualization of sable (*Martes zibellina* L. 1758) using microsatellites

**DOI:** 10.1080/19768354.2018.1494039

**Published:** 2018-07-09

**Authors:** Bo Li, Dan Wu, Yingying Cai, Monakhov Vladimir G, Wei Zhang, Yanchun Xu

**Affiliations:** a College of Wildlife Resources, Northeast Forestry University, Harbin, People's Republic of China; b State Forestry Administration Detecting Center of Wildlife Resources, Harbin, People's Republic of China; c College of Biotechnology and Engineering, West Yunnan University, Lincang, People's Republic of China; d Institute of Plant and Animal Ecology, Ural Division of RAS, Yekaterinburg, Russia

**Keywords:** Genetic individualization, sable (*Martes zibellina*), mustelidae, microsatellites

## Abstract

Genetic individualization based on non-invasive sampling is crucial for estimating the numbers of individuals in endangered mammalian populations. In sable (*Martes zibellina*)-poaching cases, identifying the number of animals involved is critical for determining the penalty. In addition, investigating animal numbers for wild sable populations requires genetic individualization when collecting several samples in neighboring regions. Microsatellites have been demonstrated to be reliable markers for individual identification. Thirty-three microsatellite loci derived from Mustelidae were selected to develop a genetic individualization method for sable. Three reference populations containing 54 unrelated sables were used to calculate allele number, allelic frequencies, and the polymorphic information content of each locus. The data were subsequently used to assess the validity of a combination of twelve loci for sable individualization. We defined twelve polymorphic loci that were easy to be amplified and genotyped. Four significant deviations from Hardy-Weinberg equilibrium were observed among the 12 loci in the three populations. The match probability of an individual from the reference populations with a random individual based on the 12 loci was 1.37 × 10^−13^. Using the combination of the twelve loci provides sufficient power to individualize sables considering the levels of microsatellite polymorphism observed. These loci were successfully applied to a case of sable poaching and provided valid evidence to determine the penalty. The genetic individualization of sable based on these loci might also be useful to investigate the numbers of animals in wild populations.

## Introduction

Sable (*Martes zibellina* L. 1758) is a circumboreal species and is widespread in the taiga forests of Eurasia, especially in Russia from the Ural Mountains to eastern Siberia; in northern Mongolia, China and North Korea; and on Hokkaido in Japan (Proulx et al. 2004). Sable is generally considered to have the most beautiful and supple pelt among martens. The pelts of sables are highly valued items in the fur trade. Since the nineteenth century, intensive hunting for sable pelts has resulted in dramatic population declines of the species in China. Several efforts, including hunting bans and the development of nature reserves, have allowed the species to recolonize much of its former range. However, the sable population in China has not completely recovered (Ma and Xu 1994; Buskirk et al. 1996; Zhang et al. 2017). To protect the species, the Chinese government has added it to the list of wildlife under special state protection and has prohibited all hunting and fur trade of the species since 1989. However, the resulting scarcity of sable pelts has increased the potential for profitable sales and stimulated poaching activities. For instance, in the winter of 2012, the police discovered a poaching case in the city of YiChun in China and seized fifteen sable pelts and five skinned sable carcasses. In this case, it was necessary to determine how many sables were involved because obtaining an accurate number of individuals is critical for determining the penalty. In addition, investigating the population numbers of wild sable populations requires a method of genetic individualization when collecting several samples in neighboring regions.

Microsatellites have been demonstrated to be reliable markers for the genetic individualization of animals (Menotti-Raymond et al. 1997; Andreassen et al. 2012). The flanking regions of microsatellite DNAs are very conservative across taxa, which allows PCR primers to be transferable to related species (Engel et al. 1996). Thus, sable individualization can be performed based on published microsatellites from other Mustelidae species, such as *Martes foina* (Basto et al. 2010), *Martes americana* (Davis and Strobeck 1998), and *Mustela vison* (Vincent et al. 2003). Kashtanov et al. (2011) demonstrated that ten microsatellite markers from other mustelid species are applicable for analyzing the genetic structure of sable populations. In this paper, we developed a method for the genetic individualization of sable using twelve microsatellite loci from related mustelid species. This method was used to successfully resolve the above-describe case and might be useful for estimating the population numbers of wild populations based on non-invasive genetic sampling techniques.

## Materials and methods

### Sampling and DNA extraction

Skin and muscle specimens of sables were collected from Northeast China and Russia. The former were from raw pelts that were donated by the Fur Specimen Museum of Northeast Forestry University. The latter were sampled from the game animals in Russia. We detected 54 sable individuals from three populations in the Lesser Khingan Mountains, Southern Far East and Vakh River in Russia. The individuals were assumed to be unrelated, since they were from different locations. Total genomic DNAs were isolated with a routine phenol: chloroform method (Sambrook et al. 1989) and quantified with the DU-640 Nucleic Acid–Protein Analysis System (Beckman Coulter) according to the user's manual.

### PCR amplification and genotyping

Thirty-three primer pairs for microsatellite loci, including 13 tetranucleotide repeat loci derived from *Martes foina* (Basto et al. 2010) and 20 dinucleotide repeat loci derived from *Martes americana* (Davis and Strobeck 1998) and *Mustela vison* (Vincent et al. 2003), were selected to amplify sable microsatellite DNAs. We added a universal M13 tag (5’-AGGGTTTTCCCAGTCACGACGTT-3’) to the 5’-end of each forward primer for use in a universal dye-labeling method (Boutin-Ganache et al. 2001). FAM or JOE M13 tag oligonucleotides were used to label the amplification products. PCR amplification was performed in a 10 µL volume containing the following: 1 × PCR buffer (10 mM Tris–HCl pH 8.3, 50 mM KCl, and 2.5 mM MgCl_2_), 0.2 mM dNTP (Takara), each of forward and reverse primer at 2.0, 1.0 ρmol fluorescently labeled M13 tag, 0.5 units of exTaq DNA polymerase (Takara), and 50–100 ng of genomic DNA. PCR amplification was performed in a Model 9700 Thermocycler (Perkin-Elmer) using the following cycling conditions: 1 cycle of 3 min at 94°C; 30 cycles of 94°C for 30 s, 54°C or 52°C for 15 s, and 72°C for 30 s; and 1 cycle of 72°C for 5 min. Amplification products were analyzed on an Applied Biosystems 3500 Genetic Analyzer (performed by Sangon Biotech, Shanghai, China), and the alleles were checked using the internal size standard GeneScan 600LIZ and GeneMapper version 5.0.

To confirm the repeat motifs of each locus in sable, we amplified all of the alleles from 5 individuals using the above-described reaction system and conditions, except that 50 µL volumes were used. Recovered PCR products were directly sequenced using the same primers on an ABI 3730 DNA analyzer (performed by BGI, Beijing, China). Consensus sequences were generated using SeqMan software (DNAStar Inc., Madison, WI, USA) and were compared to the referenced repeat sequences. The repeat number of other unsequenced alleles was later deduced in reference to their observed size and the sequenced alleles. We estimated observed heterozygosity (*H*_O_), expected heterozygosity (*H*_E_), allele number (*N*_a_), allelic frequency, polymorphism information content (PIC), and probability of identical genotypes (PI) per locus using Cervus version 3.0.3 (Kalinowski et al. 2007). Deviation from Hardy–Weinberg Equilibrium (*P_HW_*) and linkage disequilibrium were calculated using GENEPOP version 4.2.1 (Rousset 2008).

## Results

Of the 33 primer pairs examined, twenty-five pairs (75.8%) generated stable amplification products after electrophoretic analysis. We also discovered that only 13 primer pairs (39.4%) generated clear signals and were easily scored when genotyping individuals of the three populations. Through sequencing the repeat motifs of these loci, we deleted a locus (Mf 6.5) because the repeat units (TTTC) were changed to the tandem motif of TTTC and TTCC and had an insertion of C or TT, which could result in false genotyping without sequencing the alleles. The characteristics of the recognized microsatellite loci are listed in Table 1 and include seven loci from *Martes foina*, three loci from *Martes Americana* and two loci from *Mustela vison*. Eight loci presented the same motif sequences as those of the source species. There were 3 loci (Mf 2.13, Ma1, and Mvi2243) with an insertion of a 2- or 3- base motif between original repeat units and one locus (Mf 4.10) with a deletion of the initial 3-base motif among repeat units.

**Table 1.** Characteristics of the twelve microsatellite loci in sable (*N* = 54).

Locus	Repeat	Size range (bp)	*H*_o_	*H*_e_	*N*_a_	PIC	PI
Mf 1.11	(TATC)_n_	219–223	0.24	0.61	3	0.54	0.22
Mf 1.18	(ATCT)_n_	158–174	0.28	0.59	5	0.52	0.24
Mf 2.13	(TATC)_n_ATC(TATC) (TATC)_n_ATC(TATC)_3_ATC(TATC)	296–332	0.80	0.77	10	0.73	0.09
Mf 3.7	(TAGA)_4_(TGGG)(TGGA)(TAGA)_n_	165–221	0.33	0.35	7	0.33	0.44
Mf 4.10	(GAAA)_n_	276–328	0.63	0.84	13	0.82	0.04
Mf 8.7	(TCTA)_n_	137–161	0.63	0.79	7	0.75	0.08
Mf 8.8	(CTTT)_n_	230–274	0.61	0.90	12	0.88	0.02
Ma 1	(TG)_4_TA(TG)_n_	224–248	0.63	0.88	12	0.86	0.03
Ma 2	(TG)_n_	189–201	0.70	0.80	7	0.77	0.07
Ma 15	(TG)_n_	220–230	0.33	0.42	5	0.40	0.36
Mvi 354	(CA)_n_	212–232	0.80	0.85	11	0.82	0.04
Mvi 2243	(TG)_4_TA(TG)AG(TG)_n_CG(TG)_4_	169–191	0.76	0.88	12	0.86	0.03

Note: *H*_o_, observed heterozygosity; *H*_e_, expected heterozygosity; *N*_a_, allele number; PIC, polymorphic information content; PI, probability of identical genotypes per locus.

The allelic number of the twelve loci ranged from 3 to 13 (average *N*_a_ = 8.67), and the observed heterozygosity ranged from 0.24 to 0.80 (average *H*_o_ = 0.56, average *H*_e _= 0.72). Based on PIC values, Mf 8.8 was the most polymorphic locus, and Mf 3.7 was the least polymorphic locus (average PIC = 0.69). The probability of identity using the 12 loci was 1.37 × 10^−13^. Considering the existence of approximately 2.3 million sables (Monakhov 2016) in the world, use of the twelve loci together provides desirable power to individualize sables. The allelic frequencies for the total China and Russia population are shown in Table 2. The distributions of allelic frequencies of these loci were not even among the three sampled populations. A few alleles had a significantly higher frequency than the others. Tests for departures from Hardy–Weinberg (HW) equilibrium (3 populations by 12 loci) showed four significant deviations after Bonferroni correction, each of which indicated heterozygote deficiency at separate loci in different populations. Of the 198 locus pair combinations across the three populations, two pairs (1.01%) showed linkage disequilibrium (LD) after Bonferroni correction. However, as no locus combinations were consistently in linkage disequilibrium in all populations, these loci were included in the subsequent analyses.

**Table 2.** Allelic frequencies for the total China and Russia population of sables (*N *= 54).

Locus	Alleles and their frequencies (%)						
Mf 1.11	219 (52.78)	223 (27.78)	227 (19.44)				
Mf 1.18	158 (0.93)	162 (57.41)	166 (11.11)	170 (27.78)	174 (2.78)		
Mf 2.13	296 (0.93)	304 (25.93)	308 (20.37)	312 (34.26)	315 (4.63)	316 (6.48)	320 (2.78)
	327 (0.93)	328 (1.85)	332 (1.85)				
Mf 3.7	165 (0.93)	185 (11.11)	189 (79.63)	197 (4.63)	201 (1.85)	205 (0.93)	221 (0.93)
Mf 4.10	276 (0.93)	280 (33.33)	284 (6.48)	288 (9.26)	292 (7.41)	300 (5.56)	304 (0.93)
	308 (3.70)	312 (13.89)	316 (0.93)	320 (1.85)	324 (8.33)	328 (7.41)	
Mf 8.7	137 (28.70)	141 (15.74)	145 (13.89)	149 (28.70)	153 (10.19)	157 (0.93)	161 (1.85)
Mf 8.8	230 (5.56)	234 (8.33)	238 (17.59)	242 (11.11)	246 (12.04)	250 (10.19)	254 (4.63)
	258 (11.11)	262 (5.56)	266 (11.11)	270 (1.85)	274 (0.93)		
Ma 1	224 (2.78)	226 (14.81)	228 (11.11)	230 (5.56)	232 (3.70)	234 (17.59)	238 (18.52)
	240 (9.26)	242 (2.78)	244 (9.26)	246 (1.85)	248 (2.78)		
Ma 2	189 (7.41)	191 (22.22)	193 (23.15)	195 (28.70)	197 (6.48)	199 (9.26)	201 (2.78)
Ma 15	220 (75.00)	222 (8.33)	224 (1.85)	228 (6.48)	230 (8.33)		
Mvi 354	212 (2.78)	214 (1.85)	216 (5.56)	218 (12.96)	220 (4.63)	222 (6.48)	224 (12.96)
	226 (20.37)	228 (26.85)	230 (2.78)	232 (2.78)			

The amplified fragments of 12 microsatellite loci from the case evidence samples were assigned to alleles discovered in the reference population by size (Table 3). No new alleles were found in the evidence samples. Of the five skinned sable carcasses, four individuals displayed genotypes identical to those of pelt samples based on the 12 loci. These data and the PI value across all loci suggested that the five carcasses and 15 pelts were from sixteen individuals.

**Table 3.** Fragment sizes of the twelve microsatellite loci and corresponding genotypes of the evidence samples.

Locus	Observed allele size (bp) and corresponding genotype			
	B1	B2	B3	B4
Mf 1.11	222.00/222.00 219/219	222.13/226.20 219/223	222.13/230.41 219/227	222.21/222.21 219/219
Mf 1.18	161.83/161.83 162/162	161.92/161.92 162/162	161.83/161.83 162/162	161.92/165.93 162/166
Mf 2.13	303.81/312.18 304/312	303.85/316.31 304/316	308.71/315.79 308/315	311.99/327.55 312/327
Mf 3.7	189.87/189.87 189/189	189.77/189.77 189/189	189.72/189.72 189/189	189.75/201.82 189/201
Mf 4.10	285.39/292.61 284/292	281.73/281.73 280/280	289.01/289.01 288/288	281.74/281.74 280/280
Mf 8.7	148.86/153.15 149/153	144.70/148.86 145/149	148.76/152.98 149/153	140.61/144.02 141/145
Mf 8.8	241.17/241.17 238/238	245.49/253.66 242/250	232.61/245.20 230/242	236.86/241.07 234/238
Ma 1	242.27/242.27 238/238	244.47/244.47 240/240	242.29/248.63 238/244	242.22/244.35 238/240
Ma 2	198.18/198.18 197/197	196.21/196.21 195/195	192.01/196.23 191/195	190.03/202.65 189/201
Ma 15	223.00/223.00 220/220	233.34/233.34 230/230	223.36/223.36 220/220	223.2/223.2 220/220
Mvi 1354	221.73/223.63 224/226	210.02/221.86 212/224	213.73/225.58 216/228	219.77/223.68 222/226
Mvi 2243	178.66/184.90 177/183	178.59/180.62 177/179	178.71/180.67 177/179	176.75/180.82 177/179

	Observed allele size (bp) and corresponding genotype			
Locus	6	3	13	14
Mf 1.11	221.21/221.21 219/219	221.21/225.50 219/223	221.21/229.59 219/227	221.30/221.30 219/219
Mf 1.18	161.82/161.82 162/162	161.76/161.76 162/162	161.66/161.66 162/162	161.66/165.67 162/166
Mf 2.13	304.53/312.89 304/312	304.48/316.96 304/316	308.70/315.85 308/315	312.91/328.48 312/327
Mf 3.7	188.69/188.69 189/189	188.71/188.71 189/189	188.71/188.71 189/189	188.74/200.91 189/201
Mf 4.10	285.43/292.72 284/292	281.85/281.85 280/280	289.12/289.12 288/288	281.87/281.87 280/280
Mf 8.7	148.75/152.99 149/153	144.18/148.97 145/149	148.96/153.15 149/153	140.75/144.55 141/145
Mf 8.8	242.28/242.28 238/238	246.5/254.53 242/250	233.81/246.41 230/242	238.14/242.37 234/238
Ma 1	242.83/242.83 238/238	244.91/244.91 240/240	242.82/249.4 238/244	242.97/245.03 238/240
Ma 2	198.27/198.27 197/197	196.27/196.27 195/195	192.01/196.23 191/195	189.99/202.66 189/201
Ma 15	222.72/222.72 220/220	233.47/233.47 230/230	222.74/222.74 220/220	222.83/222.83 220/220
Mvi 1354	222.88/224.84 224/226	210.93/222.85 212/224	214.85/226.76 216/228	220.84/224.82 222/226
Mvi 2243	179.76/186.01 177/183	179.75/181.75 177/179	179.59/181.67 177/179	177.57/181.81 177/179

Note: B1, B2, B3 and B4 are four samples of skinned carcasses, which correspond to Nos. 6, 3, 13, and 14, respectively, of the pelt samples, which have the same genotype.

## Discussion

The cross-species amplification of polymorphic microsatellite loci in the family Mustelidae has exhibited great variability (Davis and Strobeck 1998). For example, in a study using 13 *Martes americana* microsatellite loci primers to amplify sequences in other mustelid species, the five species *Martes pennanti*, *Mustela vison*, *Lutra canadensis*, *Gulo gulo*, and *Taxidea taxus* presented amplification success rates of 76.9%, 46.2%, 30.8%, 76.9%, and 38.5%, respectively (Davis and Strobeck 1998). Since *Martes americana*, *Martes pennanti*, and *Gulo gulo* are more closely related to one another than to other mustelid species (Yu et al. 2011; Li et al. 2014), it is not unexpected that the former two species had higher success rates. In this study, we assessed the variability of 33 polymorphic microsatellite loci from *Martes foina*, *Martes americana* and *Mustela vison* in three reference sable populations. Similar results were obtained after amplification and genotyping. However, there is a risk of false genotyping when performing cross-species amplification of microsatellite loci without sequencing repeat motifs (Xu et al. 2005; Zhang 2011). Herein, we sequenced all repeat motifs of each locus to ensure correct genotyping and defined 12 polymorphic microsatellite loci for genetic individualization.

Sable comprises three extant subspecies, *Martes zibellina princeps*, *Martes zibellina linkouensis* and *Martes zibellina hamgyenensis* in northeastern China, which occur as segmented wild populations (Zhang et al. 2017). Ma and Wu (1981) argued that *Martes zibellina linkouensis* is a native subspecies with a scattered distribution limited to the Lesser Khingan Mountains and northern Zhang Guangcai Mountains in China, whereas the other two subspecies also occur in foreign neighboring areas. Li et al. (2013) highlighted the evolutionary history of sable in the southeast portion of its range based on mitochondrial DNA variation. However, the genetic background of the populations seemed very complex. Individualization using molecular genetic markers requires a database representing the species and detailing the quantitative genetic parameters (Xu et al. 2005; Andreassen et al. 2012). We sampled three populations of sable from China and Russia in this study, largely representing the whole population of northeastern China and foreign neighboring areas. The molecular genetic database strongly supported the statistical analysis of the genotyping results in criminal cases and investigations of wild populations of sable, including *Martes zibellina linkouensis*. Considering that the probability of identity based on the 12 loci combined was 1.37 × 10^−13^ and that the global population size of sable is approximately 2.3 million (Monakhov 2016), we consider the method to be valid and potentially helpful for estimating the population numbers of wild populations. However, the sample size for each subspecies was limited, and thus the microsatellite diversity was likely underestimated. Therefore, the non-invasive sampling of a larger number of individuals of each subspecies is required to improve the database.
